# Associations between Obesity, Obesogenic Environments, and Structural Racism Vary by County-Level Racial Composition

**DOI:** 10.3390/ijerph16050861

**Published:** 2019-03-09

**Authors:** Caryn N. Bell, Jordan Kerr, Jessica L. Young

**Affiliations:** 1 Department of African American Studies, University of Maryland, College Park, MD 20724, USA; 2 School of Public Health, University of Maryland, College Park, MD 20724, USA; kerr_jordan2319@yahoo.com; 3 Department of Health Studies, American University, Washington, DC 20016, USA; jessica@american.edu

**Keywords:** obesity, obesogenic environments, structural racism, racial composition

## Abstract

Obesity rates in the U.S. are associated with area-level, food-related characteristics. Studies have previously examined the role of structural racism (policies/practices that advantaged White Americans and deprived other racial/ethnic minority groups), but racial inequalities in socioeconomic status (SES) is a novel indicator. The aim of this study is to determine the associations between racial inequalities in SES with obesity and obesogenic environments. Data from 2007–2014 County Health Rankings and 2012–2016 County Business Patterns were combined to assess the associations between relative SES comparing Blacks to Whites with obesity, and number of grocery stores and fast food restaurants in U.S. counties. Random effects linear and Poisson regressions were used and stratified by county racial composition. Racial inequality in poverty, unemployment, and homeownership were associated with higher obesity rates. Racial inequality in median income, college graduates, and unemployment were associated with fewer grocery stores and more fast food restaurants. Associations varied by county racial composition. The results demonstrate that a novel indicator of structural racism on the county-level is associated with obesity and obesogenic environments. Associations vary by SES measure and county racial composition, suggesting the ability for targeted interventions to improve obesogenic environments and policies to eliminate racial inequalities in SES.

## 1. Introduction

Obesity is a health condition that predicts mortality [1,2,3], cardiovascular disease [4], and some cancers [5], and has complex determinants that range from physiological to sociocultural factors [6,7,8,9,10,11,12,13,14,15]. Obesity rates in the U.S. have grown in recent decades with some demographic groups experiencing higher prevalence rates like women, low socioeconomic status (SES), underrepresented minority groups, and rural residents [15,16,17,18,19,20,21,22,23]. Along with individual-level characteristics, various area-level factors like median income, poverty rates, and racial composition play an important role in obesity prevalence in the U.S. [17,24,25,26,27,28,29,30,31,32,33,34,35,36].

Obesogenic environments are generally characterized by having a greater number of features that promote obesity (i.e., more caloric intake and less energy expenditure), and fewer resources that promote a healthy weight [28]. For example, obesogenic environments have less healthy food access, such as fewer supermarkets and grocery stores, and more establishments with energy-dense foods such as fast food restaurants [28]. More fast food restaurants and less access to supermarkets and grocery stores has been associated with higher obesity rates [10,29,35,36]. Obesogenic environments are not evenly distributed. There is variation by area demographics, SES, urban sprawl, land use mix, and racial composition [28,37,38,39,40,41,42,43]. Lower SES neighborhoods and those that are racially segregated with disproportionately more residents who are underrepresented racial/ethnic minorities tend to be more obesogenic [37,39,42,43].

Racial residential segregation in the U.S. is an indicator of structural racism [44,45,46]. Structural racism may be defined as “the macrolevel systems, social forces, institutions, ideologies, and processes that interact with one another to generate and reinforce inequalities among racial and ethnic groups” [47]. Structural racism in the U.S. operates such that institutions and governmental systems on the federal-, state-, and local (county)-level develop, implement, and enforce laws and policies that explicitly or implicitly advantage Whites and disadvantage Blacks and other racial/ethnic minority groups [48,49,50]. For example, a history of racism in real estate practices and inequitable access to wealth-building benefits like federally-backed home loans and housing assistance available to Whites (and not Blacks) has led to disparate residential environments [51,52,53,54,55]. This form of structural racism has consistently been associated with fewer health-promoting resources, including those that promote healthy weight [11,26,42,43,51,52,56,57,58,59,60].

Racial inequalities in SES are another indicator of structural racism, but has yet to be applied to obesity and obesogenic environments. Socioeconomic status reflects an overall combination of economic and social standing, but is often measured by educational attainment, household income, occupation, and wealth [61]. Racial inequalities in SES can be considered as differences in the average level of SES between Blacks and Whites as indicated by measures such as income, education, and others. In the U.S., Blacks consistently have lower incomes [62], and are more likely to live under the poverty line [63] and to be unemployed compared to Whites [64]. Blacks are less likely to complete a four-year college degree [65], and less likely to be homeowners [66,67,68]. These social disadvantages among Blacks can be largely attributed to the racism of U.S. institutions that advantaged Whites and restricted Black opportunity [69], resulting in large race differences in average SES in the U.S. [70]. The racism of real estate institutions, and local and federal policies that led to racial segregation also contributed to racial inequality in SES [55]. Racial segregation contributes to differences in the job market and unemployment, homeownership opportunity, and disparate school systems and educational experiences that can lead to lower college graduation rates and earning potential [54,55].

Recent literature has emerged that examines area-level racial inequalities in SES (and other indicators of structural racism like unequal sentencing in the judicial system) and health [44,45,46,47,48,49,71,72]. Studies demonstrate that states and counties with larger average differences in SES indicators, such as income and educational attainment between Blacks and Whites have worse health outcomes [45,48,49,71,72], including myocardial infarctions [71], a cardiovascular disease outcome associated with obesity [4]. For example, Lukachko et al. found that states with larger relative differences in the percentage of employed adults between Blacks and Whites had higher myocardial infarction rates among Blacks. It is possible that structural racism in the form of racial inequalities in SES could be associated with obesity and obesogenic environments. One previous study has also examined the effects of area-level racial inequalities as a novel measure of structural racism and compared it with more traditional measures of structural racism like racial segregation [45]. The study found variation between novel and traditional measures of structural racism, therefore it is important to understand how the associations between area-level racial inequalities with obesity and obesogenic environments operate in light of other area-level characteristics that have been traditionally examined with regard to obesity and the food environment, such as racial composition.

The aim of this study is to assess the associations between county-level racial inequalities in SES with obesity and obesogenic environments. It is hypothesized that counties that demonstrate larger racial inequalities in SES will have higher rates of obesity, fewer supermarkets, and more fast food restaurants. Additionally, this study will compare the effects of structural racism on obesity and the food environment by county racial composition. It is hypothesized that structural racism will be associated with obesity and the food environment in counties with more Black residents compared to counties with fewer. Determining these associations will expand the understanding of the ways in which structural racism pervades health and potentially lead to addressing racial inequalities in SES and interventions to improve population health.

## 2. Methods

County Health Rankings (CHR) is compilation of health and health-related outcomes in U.S. counties over time [73]. A collaboration between the Robert Wood Johnson Foundation and the University of Wisconsin Population Health Institute, CHR collects data from various sources to develop a ranking of health based on several factors. The source of data for this study was the Behavioral Risk Factor Surveillance System (BRFSS). The BRFSS is an annual survey conducted by state-level health departments of population health status and health behaviors such as smoking, alcohol consumption, physical activity, and healthcare utilization [74]. A base survey with the option of additional modules is collected every year by state health departments and selected data are collected by CHR. Data from every U.S. county from 2007 to 2014 were included in this study. Data from the County Business Patterns (CBP) data set from the U.S. Census Bureau [75] was also utilized. Annual CBP data includes the number of every business registered in each U.S. county by category using the North American Industry Classification System (NAICS). Data from 2012 to 2016 were included. County-level health data was linked with county-level sociodemographics from the American Community Surveys 5-Year estimates (ACS) [76]. The ACS is a survey of the U.S. population that includes data on economic, geographic, and housing data from the United States, Puerto Rico, and other U.S. territories, and is conducted by the U.S. Census Bureau annually. Five years of data were compiled to obtain representative data for every U.S. county. The five-year combined estimates were represented by the last year of data in the five-year group in these data analyses.

Four dependent variables were analyzed and included obesity, the number of grocery stores and fast food restaurants, and the ratio of fast food restaurants to grocery stores in U.S. counties. BRFSS respondents were asked their height and weight, and body mass index (BMI) was calculated. The percentage of respondents in each county with a BMI ≥ 30 kg/m^2^ was calculated. Grocery stores were categorized as businesses with a NAICS code of 445110, which are defined as “establishments generally known as supermarkets and grocery stores primarily engaged in retailing a general line of food, such as canned and frozen foods, fresh fruits and vegetables, and fresh and prepared meats, fish and poultry.” The number of grocery stores in each county was included. Businesses that were categorized with a NAICS code of 722513 are described as “establishments primarily engaged in providing food services (except snack and non-alcoholic beverage bars) where patrons generally order or select items and pay before eating” were included. The number of limited service or fast food restaurants in each county were included. Recent studies find that unhealthy food resources, such as fast food restaurants and corner stores, are more predictive for obesity than access to healthy food resources like grocery stores [10,77], so it is important to include a dependent variable that compares unhealthy food resources to healthy food resources. A variable that represents the ratio of fast food restaurants to grocery stores was calculated for each county.

Five structural racism indicators representing racial inequalities in SES in U.S. counties were the independent variables. The median income, percentage living below the poverty line, percentage who completed a four-year college degree, percentage who were unemployed, and percentage who were homeowners for non-Hispanic Blacks and non-Hispanic Whites were obtained for each county. Structural racism was measured as racial inequality in these indicators, operationalized as county-level Black–White ratios. Variables were categorized such that no difference or a Black–White ratio that indicated that Blacks in that county had better outcomes (i.e., higher median income, lower percentage of poor residents, higher percentage of college graduates, lower percentage of unemployed residents, and higher percentage of homeowners) were given a value of zero to represent no racial inequality. Dummy variables to represent quartiles of racial inequality were labeled: low, medium, medium high, and high.

County racial composition was the stratifying variable. The percentage of county residents who were Black/African American was assessed. The average percentage of Black residents was assessed and a dichotomous variable was created to reflect counties where the percentage of Black residents was greater than the average. Several covariates included: county population, area, urbanization category, physical inactivity, overall median income, percentage of residents living below the poverty line, percentage of residents who completed a four-year college degree, percentage of residents who were unemployed, and percentage of residents who were homeowners. All co-variates were measured continuously with the exception of urbanization. County urbanization categories were based on the 2013 National Center for Health Statistics Urban-Rural Classification Scheme for Counties. These categories were based on population density and proximity to a metropolitan area. Categories included central metro, central fringe metro, medium metro, small metro, micropolitan, and non-core.

Differences in county characteristics, structural racism indicators, and obesity and obesogenic environments by racial composition were assessed using Student’s *t* and chi-square tests. Random effects regressions were used to determine the associations between indicators of structural racism with county-level obesity and obesogenic environments. The dataset was analyzed as panel data such that county was the panel variable and year was the time variable. Linear regressions were used when the dependent variable was obesity, and adjusted for all covariates. Poisson regressions were used when the dependent variable was number of grocery stores or fast food restaurants with county population as the exposure variable. Regression analyses were stratified to examine the associations between structural racism indicators with obesity and the food environment by racial composition. If *p*-values were less than or equal to 0.05, they were considered statistically significant, and all *t*-tests were two-sided. All statistical procedures were performed using Stata statistical software, Version 14 (StataCorp LP, College Station, TX, USA).

## 3. Results

Table 1 displays county-level demographics, indicators of structural racism, health related resources, and obesity in U.S. counties by racial composition from 2007 to 2016. County population, size, urbanization, percentage of physically inactive residents, median income, poverty rates, college graduation rates, unemployment rates, and homeownership rates varied by county racial composition. The Black–White ratio of median income demonstrated that the Black median income was lower than the White median income, but the magnitude of this difference was larger in counties with more Black residents (*p* < 0.001). There was no difference in the Black–White poverty ratio by county racial composition (*p* = 0.502). In counties that were ≤9% Black, the Black–White ratio in college graduation was 0.67 (standard error (S.E.) = 0.01). This ratio was 0.51 (S.E. = 0.01) in counties with >9% Black residents (*p* < 0.001). The racial difference in unemployment was larger in counties that were ≤9% Black compared to counties that were >9% Black (*p* = 0.011). The Black–White ratio in homeownership in counties with fewer Black residents reflected a larger racial difference compared to counties with more Black residents (*p* < 0.001). Obesity rates were lower in counties with fewer Black residents compared to those with more (28.5% versus 30.9%, *p* < 0.001). Counties that were ≤9% Black had more grocery stores per 10,000 population (27.9 ± 0.2) compared to counties with >9% Black residents (20.3 ± 0.2, *p* < 0.001). The number of fast food restaurants per 10,000 population was lower in counties that were ≤9% Black (60.8 ± 0.3) compared to counties with more Black residents (64.4 ± 0.5, *p* < 0.001). In counties with fewer Black residents, the ratio of fast food restaurants to grocery stores was lower (3.38 ± 0.02) compared to those with more Black residents (3.83 ± 0.04, *p* < 0.001).

Associations between structural racism indicators and obesity were displayed in Table 2. Medium high (β = 0.28, S.E. = 0.12) and high (β = 0.45, S.E. = 0.13) racial inequality in poverty was associated with higher obesity rates. Compared to counties with no racial inequality in college graduation, counties with medium high racial inequality in college graduation had obesity rates that were 0.31 percentage points lower (β = −0.31, S.E. = 0.12). Medium high (β = 0.37, S.E. = 0.12) and high (β = 0.28, S.E. = 0.12) racial inequality in unemployment was associated with higher obesity rates, as was low (β = 0.37, S.E. = 0.10) and medium (β = 0.23, S.E. = 0.12) racial inequality in homeownership. Counties with >9% Black residents had higher obesity rates (β = 1.10, S.E. = 0.13). The associations between structural racism and obesity in counties with ≤9% Black residents reflected closely with the overall results such that medium high (β = 0.35, S.E. = 0.13) and high (β = 0.37, S.E. = 0.13) racial inequality in poverty, low (β = 0.30, S.E. = 0.12) and medium high (β = 0.33, S.E. = 0.12) inequality in unemployment and low (β = 0.25, S.E. = 0.12) racial inequality in homeownership were associated with higher obesity rates. Medium high racial inequality in college graduation (β = −0.32, S.E. = 0.13) was associated with lower obesity rates in counties that were ≤9% Black. In counties with >9% Black residents, high racial inequality in college graduation was associated with lower obesity rates (β = −1.79, S.E. = 0.56). Medium (β = −0.85, S.E. = 0.41) and medium high (β = −1.18, S.E. = 0.42) racial inequality in homeownership was associated with lower obesity rates in counties with more Black residents.

Table 3 displays the association between structural racism indicators and grocery store density by racial composition in U.S. counties. Racial inequality in income was associated with fewer grocery stores per 10,000 population. Low (incidence rate ratio (IRR) = 0.94, 95% CI = 0.90–0.99) and medium (IRR = 0.93, 95% CI = 0.89–0.98) racial inequality in poverty was associated with fewer grocery stores. Compared to counties with no racial inequality in college graduation rates, counties with low inequality had a 5% lower rate of grocery stores. Racial inequality in unemployment and homeownership was associated with fewer grocery stores. Counties with ≥9% Black residents had 8% higher incidence rates of having a grocery store compared to counties with <9% Black. In counties with <9% Black residents, racial inequality in median income, poverty, college graduation rates, unemployment, and homeownership were associated with fewer grocery stores. In counties that were ≥9% Black, racial inequality in median income, poverty, unemployment, and homeownership were not associated with the number of grocery stores. However, compared to counties with no racial inequality, counties with low (IRR = 1.15, 95% CI = 1.02–1.30), medium (IRR = 1.21, 95% CI = 1.07–1.38), medium high (IRR = 1.26, 95% CI = 1.11–1.44), and high (IRR = 1.36, 95% CI = 1.04–1.78) racial inequality in college graduation rates had more grocery stores in counties with more Black residents.

In Table 4, the association between indicators of structural racism and fast food restaurants are displayed. Low, medium, and medium high racial inequality in median income and college graduate rates were associated with more fast food restaurants compared to counties with no racial inequality. Counties with low racial inequality in unemployment had 3% higher incidence rates of fast food restaurants (IRR = 1.03, 95% CI = 1.01–1.05) compared to counties with no racial inequality. There were fewer fast food restaurants in counties with low (IRR = 0.96, 95% CI = 0.94–0.99) and medium (OR = 0.97, 95% CI = 0.95–0.99) racial inequality in homeownership compared to counties with no racial inequality. Among counties with <9% Blacks, racial inequality in median income, college graduation rates, and unemployment were associated with more fast food restaurants, while racial inequality in homeownership was negatively associated with fast food restaurants. In counties with ≥9% Black residents, no indicators of structural racism were associated with the number of fast food restaurants.

The associations between indicators of structural racism and the ratio of fast food restaurants to grocery stores is displayed in Table 5. Compared to counties with no racial inequality in median income, those with low (β = 0.20, S.E. = 0.07), medium (β = 0.32, S.E. = 0.08), medium high (β = 0.30, S.E. = 0.07), and high (β = 0.21, S.E. = 0.08) racial inequality had higher ratios of fast food to grocery stores. Counties with medium racial inequality in poverty (β = 0.16, S.E. = 0.07) had higher fast food-to-grocery store ratios compared to counties with no poverty racial inequality. In counties with low (β = −0.19, S.E. = 0.06), medium (β = −0.24, S.E. = 0.06), and medium high (β = −0.22, S.E. = 0.06) racial inequality in homeownership, the ratio of fast food-to-grocery stores was lower compared to counties with no racial inequality in homeownership. In counties that were <9% Black, racial inequality in median income and college graduation rates were associated with higher ratios of fast food-to-grocery stores. Racial inequality in homeownership was associated with lower fast food-to-grocery store ratios in counties with <9% Black residents. In counties that were ≥9% Black, racial inequality in median income, poverty, unemployment, and homeownership were not associated with the ratio of fast food restaurants to grocery stores. However, medium (β = −0.53, S.E. = 0.23), medium high (β = −0.55, S.E. = 0.24) and high (β = −1.26, S.E. = 0.31) racial inequality in college graduation rates were associated with lower ratios of fast food restaurants to grocery stores in counties with ≥9% Black residents.

## 4. Discussion

Though a growing literature demonstrates the effects of structural racism on population health [45,47,48,49,71,72], there are gaps around the importance of structural racism to obesity and related environmental factors. This study sought to assess the association between racial inequalities in socioeconomic status as indicators of structural racism with obesity and obesogenic environments and to determine whether these associations varied by racial composition. Some, but not all, indicators of structural racism were associated with obesity, and the number of grocery stores and fast food restaurants. Controlling for county-level SES and other covariates, racial inequality in poverty, unemployment, and homeownership was associated with higher obesity rates. Structural racism as indicated by racial inequality in income, unemployment, and homeownership was associated with fewer supermarkets. Racial inequality in homeownership was associated with fewer fast food restaurants, but racial inequality in income and college graduation was associated with more fast food restaurants. In counties with fewer Black residents, the results tended to mirror the full analyses. However, in counties with more Black residents, some indicators of structural racism were associated with lower rates of obesity, more grocery stores, fewer fast food restaurants, and lower ratios of fast food-to-grocery stores. These results demonstrate the effects of structural racism on county-level obesity and obesogenic environments and further explicate the potential mechanism by examining racial composition as a possible modifying factor.

To the authors’ knowledge, no previous studies have examined the association between structural racism as indicated by racial inequality in SES on obesity and the food environment. Studies have suggested that another form of structural racism, racial segregation, is associated with higher obesity rates and more obesogenic food environments [11,26,42,43,51,52,56,57,58,59,60]. A study has previously examined the effects of area-level racial inequalities on myocardial infarction, and found that state-level racial inequalities in employment were associated with higher rates of myocardial infarction among Blacks, but no association among Whites [71]. There was no association between racial inequalities in college graduation rates and myocardial infarctions among neither Blacks nor Whites [71]. The results of the current study found that obesity was positively associated with racial inequalities in unemployment, but negatively associated with racial inequalities in college graduation. Differences in results in the current study and the study by Lukachko et al. could be due to geographic level of analyses (i.e., county-level versus state-level) and analyses of race-specific outcomes in the Lukachko study [71].

Higher obesity rates in counties with racial inequalities in poverty, employment, and homeownership suggest that these social contexts are more obesogenic. The results of the current study suggest that these environments tend to have fewer grocery stores and more fast food restaurants. However, the social environments of these contexts may be associated with higher obesity rates. The social environment affects obesity rates [78], and scholars have posited that one of the important potential characteristics of areas in which structural racism is observed through racial inequalities in SES is that these contexts may be characterized by higher levels of stress [71,72]. Stress and stressful environments are cited as predictors of poorer health and unhealthy behaviors [79,80,81,82,83]. Scholars have applied ecosocial theory with regard to the effects of structural racism on health such that the social context is embodied in individuals and their health [45,71]. More stressful environments may be associated with poor dietary behaviors as a form of stress coping, and thus are associated with higher obesity rates.

Contexts in which racial inequalities are experienced may have a higher prevalence of other stress-related characteristics such as higher crime rates [84] or less social cohesion. Observing and experiencing inequalities between social groups could lead to a stress-inducing cognitive dissonance due to a mismatch between societal ideals of equality and the racial inequalities that are observed [46]. These stressful social contexts may lead to poor health behaviors, such as overconsumption of high caloric, stress-relieving foods, to cope with the racial inequalities observed.

There were fewer grocery stores, more fast food restaurants, and a higher ratio of fast food restaurants to grocery stores in counties with higher racial inequalities in median income, poverty, and unemployment. The allocation of these types of resources may be a response to consumer demands. Counties with larger racial inequalities may experience economic demands for more fast food restaurants and fewer grocery stores because of the consumer environment that responds to the need to cope with the more stressful environment of counties in which racial inequalities in socioeconomic status are larger. Previous studies have found that low-income and minority communities are targeted by unhealthy food resources [43,85,86]. It is unknown whether areas with large racial inequalities in SES are targeted similarly. However, other factors like zoning and political activism can be important to the distribution of food-related resources such as grocery stores and fast food restaurants. Counties with larger racial inequalities may be characterized by less political and social efficacy that could be associated with fewer grocery stores and more fast food restaurants. That is, residents of counties with large racial inequalities may not have sufficient sociopolitical power to obtain a higher number of grocery stores and reduce the number of fast food restaurants.

This explanation may be supported by the results that find that racial inequalities in SES like college graduation and homeownership are associated with better outcomes, particularly in counties with more Black residents. The results demonstrate that counties with racial inequalities in homeownership have fewer fast food restaurants and a lower fast food-to-grocery store ratio, while counties with larger racial inequalities in college graduation rates had lower obesity rates. These counties may be characterized by particularly high rates of homeownership among Whites and lower rates among Blacks that may correspond with high sociopolitical efficacy among Whites such that, by whatever means, residents in these counties may be able to demand fewer fast food restaurants. Studies find that college education is more strongly associated with lower obesity rates among Whites than Blacks [87,88]. Counties with very high rates of White college graduates relative to Black college graduates may have lower rates of obesity overall because of much lower rates of obesity among Whites who, with higher education levels [9,89,90], may have access to health-promoting resources like grocery stores and recreational facilities.

However, the potential benefits of high racial inequality in homeownership and college graduation in counties to obesity and the food environment may not be experienced by Blacks living in those counties. The results found that, in counties with higher than average concentrations of Black residents, higher racial inequality in college graduation rates was associated with lower rates of obesity, more grocery stores, and lower fast food-to-grocery store ratios. In these counties with more Black residents, racial inequality in homeownership was associated with lower obesity rates. These counties may have more Black residents, but racial groups may be spatially segregated such that there is unequal access to health-promoting resources that may lead to lower obesity rates among those who live in areas with more grocery stores and fewer fast food restaurants. Black residents may also be segregated into areas in these counties that have more stress-inducing characteristics and Whites may experience relatively less exposure to stress. This may also play a role in lower obesity rates among Whites in counties with larger racial inequalities in SES. Though the current study did not assess the role of racial residential segregation, scholars have discussed structural racism in terms of the ubiquity and pervasiveness of social advantages for Whites to the point that “color-blind” policy results in disadvantages for Blacks and that these are a permanent part of U.S. society [46,91,92]. Racial residential segregation and racial inequalities in SES may be linked and affect the association between racial inequalities in SES with obesity and obesogenic environments.

Harrell et al. (2011) posit that the pathways between structural racism and health include “classically conditioned associations between race and negative circumstances” [46]. Implicit bias and subconscious stereotypes toward Blacks and racist cognitive schema [46] may be more prevalent in areas where racial inequalities in SES are larger. However, these mechanisms likely differ by race. Scholars suggest that structural racism may be associated with poorer health outcomes through maladaptive coping behaviors, stereotype threat, worry, and rumination [46,49]. For example, Blacks who live in areas with larger racial inequalities in SES may experience stereotype threat, where there is fear and worry about fulfilling the societal stereotypes of that racial group. This type of response could be associated with higher chronic stress and poorer dietary behaviors to cope with this stress [79,80,81,82,83]. Racist cognitive schema may also affect stress coping behaviors in Blacks. Though Harrell et al. (2011) suggest interventions to address the effects of cultural racism on racist cognitive schema and health among Blacks, addressing racist stereotypes about Blacks in counties with larger racial inequalities in SES may be associated with obesogenic environments. Racist stereotypes that may accompany large racial inequalities in SES may affect the factors that are linked to the allocation of grocery stores and fast food restaurants such as perceptions of consumer demands.

Addressing the effects of and dismantling the systemic characteristics of structural racism that lead to inequalities in SES is a social justice imperative in the U.S. Racial inequalities in SES and other social outcomes are the result of consistent and pervasive efforts among national-, state-, and county-level institutions to advantage Whites and disadvantage Blacks and other racial/ethnic groups [44,47,48,49,50,54,55]. Most of the results demonstrate that racial inequalities in SES are associated with worse outcomes, so beyond the social justice imperative, structural racism has public health implications. Though racial inequalities in SES were associated with better outcomes in some instances, it is likely that these benefits are not equally distributed to the disadvantaged Blacks in those counties in which large racial inequalities were associated with lower rates of obesity, more grocery stores and lower ratios of fast food restaurants to grocery stores. Whatever the potential health benefits of structural racism, they should not supersede the necessity of addressing the racial inequalities due to structural racism.

Policymakers have demonstrated an increased interest in reducing obesity and addressing obesogenic environments in the U.S. [93]. To reduce racial inequalities in obesity and obesogenic environments, policymakers should systematically incorporate measures of structural racism and related racial inequalities into their decision-making processes. Policymakers and their staff could integrate measures of structural racism beyond racial segregation, such as racial inequalities in income, poverty, homeownership, and education into their decision-making processes using race equity impact assessments and other tools. One tool that policymakers can use is “racial impact statements.” These statements are similar to fiscal or environmental impact statements and would allow policymakers to assess unjust racial inequalities that may arise with particular legislation and other policy-related decisions. Racial impact statements may also enable policymakers to consider alternative approaches to accomplish desired goals without fostering inequitable racial impacts [94]. This approach has been applied to criminal justice and incarceration policies and could be adapted to policies to address racial inequalities in obesogenic environments.

Among the many strengths of this study was that it was nationally representative and included multiple years of data. The study was also able to assess structural racism on the county-level. Scholars suggest the importance of the geographical level of analysis [44], and that county-level is the unit of analysis at which governance power and allocation of resources is often conducted [45]. There are some limitations to the study. Because of the cross-sectional design, a causal relationship could not be assessed. Structural racism in the U.S. is ubiquitous and is observed in many areas of society. Previous studies that have examined associations between structural racism in the form of racial inequalities and health have operationalized structural racism as racial relative ratios of SES as well as racial differences in experiences with the judicial and political system. The current study was not able to include these types of measures because of the difficulty in obtaining measures of structural racism in judicial and political systems for all counties in the U.S. for multiple years. The study also could not assess race-specific outcomes. Obesity in this data set represents the overall percentage of county residents with BMI ≥ 30 kg/m^2^. It is possible that associations between county-level racial inequalities in SES with obesity among Blacks could differ from the associations among Whites. Previous studies have demonstrated that structural racism is associated with myocardial infarctions and infant outcomes among Blacks, but not Whites [45,48,71]. Obesity was based on self-reported data. The study was unable to determine the associations between obesogenic environments and obesity because of a mismatch in the years in which the data were collected. The study only determined the association between racial inequalities in SES and overall number of grocery stores and fast food restaurants per 10,000 population in a county. The study was unable to ascertain the role of racial segregation and the potential racial differences in access to these resources. The study was unable to account for the role of physical activity resources on obesity. Lastly, the study is limited by the manner in which grocery stores and fast food restaurants were defined. Though those businesses included as grocery stores were described as those “primarily engaged in retailing a general line of food,” it is possible that these establishments may not be a source of healthy foods like fresh fruits and vegetables or the quality of these healthy foods may be comparatively low. The definition for fast food restaurants could also include establishments at which customers are able to obtain healthy food options such as salads. It is possible that individuals may choose healthy food options at fast food restaurants. It is also possible that individuals may purchase unhealthy foods at grocery stores.

## 5. Conclusions

In light of these limitations, this study demonstrates that structural racism operationalized as racial inequalities in SES at the county-level is associated with higher rates of obesity, fewer grocery stores, more fast food restaurants, and a higher fast food-to-grocery store ratio. The associations varied by SES measure and by county racial composition. Future studies should seek to determine how racial inequalities in SES may differentially affect obesity and obesogenic environments among Blacks and Whites. Policymakers and social justice advocates should seek to ameliorate racial inequalities in SES and address the obesogenic environment in these areas. Public health practitioners should target higher obesity rates in counties where large racial inequalities in poverty, unemployment, and homeownership are present.

## Figures and Tables

**Table 1:** County-level demographics, health–related resources, and indicators of structural racism by urbanization in the U.S. from 2007–2016.

	≤9% Black	>9% Black	*t*	*p*-Value
	*n* = 19,358	*n* = 7270		
% Black, mean ± S.E.	1.9 ± 0.0	27.5 ± 0.2	−0.01	<0.001
Population, mean ± S.E.	82751 ± 2195	187239 ± 4791	−22.56	<0.001
Area (square miles), mean ± S.E.	1434 ± 31	643 ± 7	15.38	<0.001
Urbanization category, %				
Central metro	0.8	7.3		<0.001
Central fringe metro	11.0	16.6		
Medium metro	10.9	17.3		
Small metro	11.1	14.1		
Micropolitan	21.9	15.6		
Non-core	44.4	29.3		
% physically inactive, mean ± S.E.	26.5 ± 0.0	28.8 ± 0.1	−27.02	<0.001
Median income ($10,000), mean ± S.E.	4.69 ± 0.01	4.38 ± 0.02	18.80	<0.001
% living in poverty, mean ± S.E.	13.5 ± 0.1	17.9 ± 0.1	−48.20	<0.001
% college graduate, mean ± S.E.	13.3 ± 0.1	12.6 ± 0.1	9.26	<0.001
% unemployed, mean ± S.E.	7.3 ± 0.1	9.9 ± 0.0	−53.49	<0.001
% homeowner, mean ± S.E.	73.3 ± 0.1	68.9 ± 0.1	41.07	<0.001
Income inequality, mean ± S.E.	0.74 ± 0.01	0.58 ± 0.01	32.22	<0.001
Poverty inequality, mean ± S.E.	2.69 ± 0.03	2.66 ± 0.03	0.67	0.502
College graduation inequality, mean ± S.E.	0.67 ± 0.01	0.51 ± 0.01	13.86	<0.001
Unemployment inequality, mean ± S.E.	2.60 ± 0.05	2.38 ± 0.03	2.54	0.011
Homeownership inequality, mean ± S.E.	0.62 ± 0.01	0.69 ± 0.01	−13.80	<0.001
% obese, mean ± S.E.	28.5 ± 0.1	30.9 ± 0.1	−19.87	<0.001
Grocery stores (per 10,000 population), mean ± S.E.	27.9 ± 0.2	20.3 ± 0.2	18.91	<0.001
Fast food restaurants (per 10,000), mean ± S.E.	60.8 ± 0.3	64.4 ± 0.5	−6.42	<0.001
Fast food—grocery store ratio, mean ± S.E.	3.38 ± 0.02	3.83 ± 0.04	−10.49	<0.001

Note: S.E. = standard error.

**Table 2:** Associations between indicators of structural racism and obesity by racial composition in U.S. county-years, 2007–2014.

		≤9% Black	>9% Black
	β (S.E.)	β (S.E.)	β (S.E.)
Income			
None	---	---	---
Low	−0.19 (0.10)	−0.18 (0.11)	0.10 (0.59)
Medium	−0.06 (0.11)	−0.02 (0.12)	0.06 (0.63)
Medium high	−0.18 (0.12)	−0.13 (0.13)	−0.14 (0.63)
High	−0.07 (0.11)	−0.10 (0.11)	−0.01 (0.65)
Poverty			
None	---	---	---
Low	0.14 (0.11)	0.18 (0.11)	0.05 (0.43)
Medium	0.13 (0.12)	0.15 (0.13)	0.17 (0.45)
Medium high	0.28 (0.12) *	0.35 (0.13) *	0.17 (0.47)
High	0.45 (0.13) *	0.37 (0.13) *	0.66 (0.50)
College graduation			
None	---	---	---
Low	−0.12 (0.10)	−0.13 (0.11)	−0.14 (0.35)
Medium	−0.04 (0.11)	−0.03 (0.13)	−0.31 (0.36)
Medium high	−0.31 (0.12) *	−0.32 (0.13) *	−0.62 (0.38)
High	−0.17 (0.13)	−0.09 (0.13)	−1.79 (0.56) *
Unemployment			
None	---	---	---
Low	0.19 (0.10)	0.30 (0.12) *	0.11 (0.25)
Medium	0.19 (0.11)	0.21 (0.12)	0.23 (0.27)
Medium high	0.37 (0.12) *	0.33 (0.12) *	0.41 (0.30)
High	0.28 (0.12) *	0.21 (0.13)	0.38 (0.35)
Homeownership			
None	---	---	---
Low	0.37 (0.10) *	0.25 (0.12) *	−0.63 (0.42)
Medium	0.23 (0.10) *	0.17 (0.12)	−0.85 (0.41) *
Medium high	0.06 (0.10)	0.12 (0.11)	−1.18 (0.42) *
High	0.07 (0.10)	0.12 (0.11)	−0.52 (0.47)
>9% Black	1.10 (0.13) *	---	---

Notes: * *p* < 0.05. S.E. = standard error. Analyses adjusted for racial composition; overall county median income; poverty, college graduation, unemployment, and homeownership rates; and urbanization.

**Table 3:** Associations between indicators of structural racism and grocery stores by racial composition in U.S. county-years, 2012–2016.

		≤9% Black	>9% Black
	IRR (95% CI)	IRR (95% CI)	IRR (95% CI)
Income inequality			
None	1.00	1.00	1.00
Low	0.90 (0.87–0.93)	0.91 (0.88–0.95)	1.07 (0.83–1.38)
Medium	0.87 (0.84–0.91)	0.90 (0.86–0.93)	1.02 (0.79–1.32)
Medium high	0.87 (0.83–0.90)	0.89 (0.86–0.93)	1.02 (0.79–1.32)
High	0.89 (0.85–0.93)	0.90 (0.86–0.94)	1.08 (0.83–1.40)
Poverty inequality			
None	1.00	1.00	1.00
Low	0.94 (0.90–0.99)	0.95 (0.91–0.99)	0.89 (0.72–1.10)
Medium	0.93 (0.89–0.98)	0.95 (0.90–0.99)	0.91 (0.73–1.12)
Medium high	0.96 (0.92–1.01)	0.97 (0.92–1.02)	0.93 (0.75–1.16)
High	0.99 (0.94–1.04)	0.99 (0.94–1.04)	0.96 (0.77–1.19)
College graduation inequality			
None	1.00	1.00	1.00
Low	0.95 (0.92–0.99)	0.95 (0.92–0.99)	1.15 (1.02–1.30)
Medium	0.97 (0.93–1.01)	0.97 (0.93–1.01)	1.21 (1.07–1.38)
Medium high	0.98 (0.94–1.03)	0.96 (0.92–1.01)	1.26 (1.11–1.44)
High	1.04 (0.99–1.09)	1.02 (0.93–1.07)	1.36 (1.04–1.78)
Unemployment inequality			
None	1.00	1.00	1.00
Low	0.94 (0.91–0.97)	0.96 (0.90–1.00)	0.98 (0.86–1.12)
Medium	0.93 (0.90–0.97)	0.94 (0.91–0.98)	1.00 (0.87–1.14)
Medium high	0.94 (0.91–0.98)	0.95 (0.91–0.99)	0.98 (0.85–1.13)
High	0.97 (0.93–1.01)	0.96 (0.92–1.01)	0.99 (0.85–1.17)
Homeownership inequality			
None	1.00	1.00	1.00
Low	0.94 (0.91–0.97)	0.94 (0.90–0.98)	0.86 (0.60–1.21)
Medium	0.95 (0.92–0.98)	0.96 (0.93–0.99)	0.86 (0.61–1.21)
Medium high	0.96 (0.93–0.99)	0.97 (0.94–0.99)	0.86 (0.61–1.21)
High	0.97 (0.93–0.99)	0.97 (0.93–1.01)	0.88 (0.62–1.24)
>9% Black	1.08 (1.04–1.12)	---	---

Notes: IRR = incidence rate ratio. Analyses adjusted for racial composition; overall county median income; poverty, college graduation, unemployment, and homeownership rates; urbanization; and area.

**Table 4:** Associations between indicators of structural racism and fast food by racial composition in U.S. county-years, 2012–2016.

		≤9% Black	>9% Black
	IRR (95% CI)	IRR (95% CI)	IRR (95% CI)
Income inequality			
None	1.00	1.00	1.00
Low	1.03 (1.01–1.05)	1.03 (1.01–1.06)	1.08 (0.91–1.27)
Medium	1.03 (1.01–1.05)	1.03 (1.01–1.06)	1.08 (0.91–1.28)
Medium high	1.03 (1.01–1.06)	1.04 (1.01–1.07)	1.07 (0.90–1.28)
High	1.00 (0.98–1.03)	1.00 (0.98–1.03)	1.05 (0.88–1.25)
Poverty inequality			
None	1.00	1.00	1.00
Low	0.99 (0.95–1.02)	0.99 (0.95–1.02)	0.99 (0.88–1.12)
Medium	1.00 (0.96–1.03)	0.99 (0.96–1.02)	1.03 (0.91–1.16)
Medium high	1.01 (0.97–1.04)	1.00 (0.07–1.04)	1.04 (0.92–1.18)
High	1.00 (0.96–1.03)	1.00 (0.97–1.04)	1.00 (0.88–1.13)
College graduation inequality			
None	1.00	1.00	1.00
Low	1.02 (1.01–1.04)	1.02 (1.01–1.04)	1.05 (0.99–1.13)
Medium	1.03 (1.01–1.05)	1.03 (1.01–1.05)	1.06 (0.99–1.13)
Medium high	1.03 (1.01–1.06)	1.03 (1.00–1.05)	1.08 (1.00–1.16)
High	1.00 (0.97–1.04)	0.99 (0.96–1.03)	1.00 (0.83–1.21)
Unemployment inequality			
None	1.00	1.00	1.00
Low	1.03 (1.01–1.05)	1.03 (1.01–1.06)	1.06 (0.97–1.16)
Medium	1.02 (0.99–1.04)	1.01 (0.98–1.03)	1.08 (0.99–1.19)
Medium high	1.01 (0.98–1.03)	1.00 (0.97–1.02)	1.06 (0.96–1.17)
High	0.98 (0.96–1.01)	0.99 (0.96–1.02)	1.01 (0.90–1.12)
Homeownership inequality			
None	1.00	1.00	1.00
Low	0.96 (0.94–0.99)	0.98 (0.96–1.01)	1.12 (0.90–1.41)
Medium	0.97 (0.95–0.99)	0.97 (0.95–0.99)	1.14 (0.91–1.43)
Medium high	0.98 (0.96–1.00)	0.98 (0.96–1.01)	1.16 (0.92–1.45)
High	0.99 (0.97–1.02)	1.00 (0.97–1.02)	1.15 (0.91–1.44)
>9% Black	1.00 (0.98–1.02)	---	---

Notes: IRR = incidence rate ratio. Analyses adjusted for racial composition; overall county median income; poverty, college graduation, unemployment, and homeownership rates; urbanization; and area.

**Table 5:** Associations between indicators of structural racism and fast food / grocery store ratio by racial composition in U.S. county-years, 2012–2016.

		≤9% Black	>9% Black
	β (S.E.)	β (S.E.)	β (S.E.)
Income inequality			
None	---	---	---
Low	0.20 (0.07) *	0.17 (0.07) *	0.05 (0.28)
Medium	0.32 (0.08) *	0.28 (0.08) *	0.22 (0.32)
Medium high	0.30 (0.07) *	0.25 (0.08) *	0.25 (0.31)
High	0.21 (0.08) *	0.20 (0.09) *	0.16 (0.32)
Poverty inequality			
None	---	---	---
Low	0.12 (0.07)	0.07 (0.07)	0.36 (0.26)
Medium	0.16 (0.07) *	0.13 (0.08)	0.30 (0.27)
Medium high	0.11 (0.08)	0.05 (0.09)	0.29 (0.28)
High	0.01 (0.09)	0.03 (0.10)	−0.02 (0.29)
College graduation inequality			
None	---	---	---
Low	0.06 (0.06)	0.04 (0.06)	−0.29 (0.22)
Medium	0.09 (0.07)	0.16 (0.08) *	−0.53 (0.23) *
Medium high	0.06 (0.07)	0.10 (0.08)	−0.55 (0.24) *
High	−0.15 (0.07)	−0.10 (0.08)	−1.26 (0.31) *
Unemployment inequality			
None	---	---	---
Low	0.04 (0.06)	0.02 (0.07)	0.05 (0.17)
Medium	0.09 (0.07)	0.06 (0.08)	0.05 (0.18)
Medium high	0.05 (0.07)	0.08 (0.08)	−0.04 (0.21)
High	−0.07 (0.07)	−0.03 (0.08)	−0.19 (0.21)
Homeownership inequality			
None	---	---	---
Low	−0.19 (0.06) *	−0.13 (0.08)	0.05 (0.46)
Medium	−0.24 (0.06) *	−0.20 (0.09) *	0.05 (0.46)
Medium high	−0.22 (0.06) *	−0.20 (0.07) *	0.05 (0.47)
High	0.07 (0.06)	−0.20 (0.07) *	−0.16 (0.47)
>9% Black	−0.03 (0.09)	---	---

Notes: * *p* < 0.05. S.E. = standard error. Analyses adjusted for racial composition; overall county median income; poverty, college graduation, unemployment, and homeownership rates; urbanization; and area.
